# Customizable ligand exchange on the surface of gold nanotriangles enables their application in LSPR-based sensing†

**DOI:** 10.1039/d4na00352g

**Published:** 2024-09-05

**Authors:** Ekaterina Podlesnaia, Sarmiza Elena Stanca, Buşra Çinçin, Gabriel Zieger, Andrea Csáki, Wolfgang Fritzsche

**Affiliations:** a Department of Nanobiophotonics Leibniz Institute of Photonic Technology (Leibniz-IPHT), Member of the Leibniz Research Alliance – Leibniz Health Technologies Albert-Einstein-Straße 9 07745 Jena Germany wolfgang.fritzsche@leibniz-ipht.de; b Quantum Detection Department Leibniz Institute of Photonic Technology (Leibniz-IPHT), Member of the Leibniz Research Alliance – Leibniz Health Technologies Albert-Einstein-Straße 9 07745 Jena Germany

## Abstract

Nanomaterials made of noble metals have been actively utilized in sensorics and bioanalytics. Nanoparticles of anisotropic shapes are promising for increasing sensitivity due to the generated hotspots of electron density. Such structures can be effectively manufactured by a relatively accessible colloidal synthesis. However, the shape control requires the attachment of a surfactant on specific crystal facets during their growth. Commonly used cetrimonium halides form a closely packed bilayer, lowering the surface accessibility for subsequent (bio)functionalization steps. While there are numerous studies on functionalizing gold nanospheres, novel materials, such as nanotriangles (AuNTs), often require thorough studies to adapt the existing procedures. This is mainly caused by the incomplete characterization of initial nanoparticle colloids in empirically developed protocols. Herein, we report a rational approach utilizing the surface area of AuNTs as a function of both their dimensions and concentration, determined with an express UV–VIS analysis. We demonstrate its efficiency for the exchange of cetyltrimethylammonium chloride (CTAC) with polystyrene sulfonate (PSS) and with biocompatible citrate using direct and indirect methods, respectively. Fourier-transform infrared spectroscopy unequivocally proves the ligand exchange. Such functionalization allows evaluating the bulk refractive index sensitivity of AuNTs as a measure of their potential in LSPR-based sensing.

## Abstract 2

Direct and indirect methods are reported to exchange a surfactant (CTAC) on the surface of gold nanotriangles, enabling applications in LSPR-based sensing.

## Graphical abstract

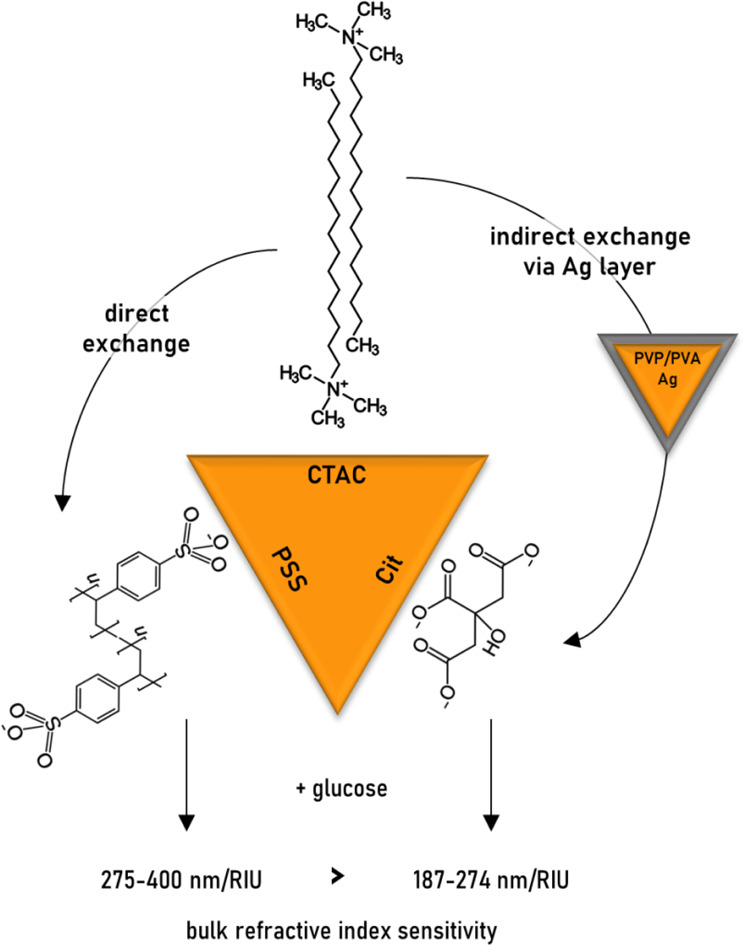

## Introduction

In the last few years, nanomaterials made of noble metals have been actively utilized for sensorics.^1–4^ Thanks to the phenomenon of localized surface plasmon resonance (LSPR) such nanoparticles respond to changes in the surrounding medium by an alteration of their spectroscopic characteristics and act as optical signal transducers.^5–7^ This allows designing compact point-of-care devices for a broad variety of bioanalytical applications.^8–13^ Gold nanoparticles (AuNPs) are known for their long-term stability in both air and solution phases in contrast to silver or copper.^14–16^ Meanwhile, nanoparticles of anisotropic shapes have drawn special interest. Sharp tips, corners, and vertices of such morphologies as rods, cubes, and triangles show an electromagnetic field enhancement providing an increase in sensitivity in various application fields,^17–21^ namely LSPR-based sensing^8,22^ or surface enhanced Raman spectroscopy (SERS).^23–27^

Among numerous methods to obtain nanomaterials, colloidal synthesis provides broad flexibility in the nanoparticle material, size and shape at relatively low manufacturing costs. The resulting colloidal solution has to be stabilized against aggregation, which is commonly realized by the use of ligands,^28,29^*e.g.* citrate. Besides the standard spherical particles derived from isotropic growth, novel protocols have been developed to synthesize anisotropic shapes. For this, surfactants such as cetrimonium halides are commonly utilized to cap selected crystal facets and hence promote shape-controlled crystal growth.^27,30,31^ While the amino group of a cetrimonium halide molecule is attached to the nanoparticle surface, the hydrocarbon tail remains insolubilized, which causes the adsorption of another surfactant layer and the formation of a close-packed bilayer.^32–34^ Being beneficial for the selected shape formation, the densely packed bilayer of the surfactant, on another hand, hinders the nanoparticle surface from further interacting with target molecules.^35^ In order to increase the accessibility or attach the required functional groups the surfactant needs to be exchanged with a different ligand. In the case of both molecules having similar charges or the use of a strongly binding ligand, such as mercaptans, also called thiols, the exchange can be performed directly by mixing an excessive amount of the required capping agent.^36–39^ However, the direct method tends to fail when the ligands carry opposite charges due to their neutralization by each other. This attenuates electrostatic repulsion, hence reducing the colloidal stability and leading to nanoparticle aggregation, especially when the target ligand is not providing enough steric stabilization.^40^ Such an approach is also not applicable when the initial capping agent has higher affinity for the gold surface compared to the required molecule.^40,41^ For these reasons, it is not possible to exchange cetrimonium halides for *e.g.* citrate in a conventional one-step method. In contrast, indirect exchange consists of multiple steps using an intermediate ligand and phase transfer, which helps remove the strongly binding molecules from the nanoparticle surface.^40,41^

Gold nanospheres and nanorods are the most commonly studied materials for which there are numerous studies in the field of surface chemistry.^28,42–44^ Meanwhile, the synthesis of such novel materials as gold nanocubes, or especially nanotriangles, has only recently been established. The existing literature is mainly focused on the simplest ligand exchange with thiol-modified molecules, which usually inevitably block the surface from further interactions.^45–48^ Most of the published procedures on surface functionalization are rather empiric and focus on a single synthesis route reporting only a few spectral characteristics as quantification parameters for nanoparticle concentration.^37,38,44,49–52^ This causes misinterpretations and makes the existing protocols rarely possible to reproduce. More recent studies refer to the concentration of gold in nanoparticle colloids.^29,40,47^ Nevertheless, various materials at the same nanoparticle concentration may have different gold content as well as surface area available for functionalization. This makes such protocols rigidly fixed to colloids with specific particle shapes and dimensions and hardly adaptable for novel materials.

Our work aims to develop systematic but versatile techniques in the area of nanoparticle surface chemistry hence facilitating downstream applications. We report a rational approach that can be customized for a variety of gold nanotriangles and potentially adjusted to other morphologies by only changing the geometry formulae. We consider the surface area as the key factor for the estimation of the required ligand amount. Being a function of nanoparticle dimensions and concentration it keeps both parameters always accounted and helps to avoid misinterpretations. At first, we describe an express method to estimate the surface area of gold nanotriangles based on the data from ultraviolet–visible (UV–VIS) spectroscopy. It utilizes the position of the LSPR peak to determine the AuNT size and the absorbance at 400 nm to estimate the concentration of metal gold (Au^0^) as in the earlier reported studies.^53–56^ This allows calculating the concentration and the total surface area in the dispersion of AuNTs together with the required amount of the ligand. We show this approach successfully working for the direct exchange of the cationic surfactant cetyltrimethylammonium chloride (CTAC) with anionic polyelectrolyte polystyrene sulfonate (PSS) on the surface of AuNTs with varied sizes (edge lengths from 30 to 80 nm). In a similar way we adapt the protocol for the indirect exchange of CTAC with citrate using the deposition and subsequent etching of an ultrathin silver layer.^40^ In this method polyvinylpyrrolidone (PVP) is used as an intermediate neutral polymer providing steric stabilization. Moreover we introduce polyvinyl alcohol (PVA) into the process for additional stabilization improving the reproducibility.^57^ The procedure efficiency is controlled by UV–VIS spectroscopy, dynamic light scattering (DLS), zeta-potential measurements, and Fourier-transform infrared spectroscopy (FTIR).

Finally, we demonstrate the potential of AuNTs in LSPR-based sensing using an express test with glucose. It serves to evaluate the bulk refractive index sensitivity (*S*_B_, nm/RIU), which shows the strength of the plasmonic response (as LSPR peak shift) to the refractive index (RI) change of the medium. This parameter is known to depend on multiple factors such as size, shape and the material composition of the nanoparticles.^58,59^ Typically, larger nanoparticles with anisotropic morphology demonstrate higher sensitivity values than smaller and/or isotropic nanoparticles. We compare the obtained data with reference values of earlier studied gold nanoparticles with various morphologies to conclude the high sensitivity of the manufactured nanotriangles. Moreover, we show *S*_B_ to be highly affected solely by the change in the surface coating within the frame of a single experiment.

## Experimental

### Materials and methods

#### Chemicals and materials

All utilized chemicals were obtained commercially and used without further purification. Tetrachloroauric(iii) acid trihydrate (HAuCl_4_·3H_2_O, ≥99.5%), l(+)-ascorbic acid (AA, ≥99%), silver nitrate (AgNO_3_, ≥99.9% p.a.), and d(+)-glucose (p.a., ACS, anhydrous) were purchased from Carl Roth GmbH & Co KG (Karlsruhe, Germany). Hexadecyltrimethylammonium chloride (CTAC, >99%) implemented in the synthesis procedure was obtained from Molekula Group GmbH (Munich, Germany). Hexadecyltrimethylammonium chloride (CTAC, 25% w/w in water) implemented in the purification procedure, sodium borohydride (NaBH_4_, 99.99%), sodium iodide (NaI, ≥99.5%), poly(sodium 4-styrenesulfonate) (PSS, average *M*_w_ ≈ 1 000 000 g mol^−1^), polyvinylpyrrolidone (PVP, average *M*_w_ 10 000 g mol^−1^), poly(vinyl alcohol) (PVA, average *M*_w_ 30 000–70 000 g mol^−1^, 87–90% hydrolyzed), and sodium citrate tribasic dehydrate (Na_3_Cit·2H_2_O, BioUltra, ≥99.5% NT) were purchased from Sigma-Aldrich Chemie GmbH (Steinheim, Germany). Hydrogen peroxide (H_2_O_2_, 30% aqueous solution, stabilized for synthesis, assay 29.0–31.0%) was received from Merck Shuchardt OHG (Hohenbrunn, Germany). Acetone (AnalaR NORMAPUR® ACS, Reag. Ph. Eur., ≥99.8%) was purchased from VWR BDH Chemicals (Fontenay-sous-Bois-cedex, France). The solutions were prepared using ultrapure Milli-Q water (EQ 7000; Merck KGaA, Darmstadt, Germany).

#### Synthesis of CTAC-capped AuNTs

The colloids of CTAC-capped AuNTs were derived using the seed-mediated method according to the earlier reported protocol.^54^ Two sample series were prepared utilizing 300, 100, 80, 60, and 40 μL of intermediate seeds (*V*_IS_ in Tables S1 and S3†) to obtain nanoparticles of various sizes for direct and indirect ligand exchange of CTAC with PSS and citrate, respectively (Tables S1 and S3:† AuNT edge length *L*_NT_ varied from 34 to 74 nm).

#### Direct exchange of CTAC with PSS on the AuNT surface

3.8 mL of the as-synthesized CTAC-capped AuNTs were centrifuged two times and re-dispersed in 5.0 mL of 0.1 mM CTAC in order to remove excessive surfactant molecules. The calculated amount of PSS (Table S1†) was dissolved in 4.0 mL of ultrapure H_2_O and added to nanoparticle colloids. After incubation for 8 h under agitation at RT, the mixtures were centrifuged two times using 0.01 mM PSS for washing. The precipitates were finally re-dispersed in 0.15 mL of 0.01 mM PSS. The molarity of PSS here is based on the molecular weight of its monomeric unit *M*_m.u._ = 206.20 g mol^−1^.

#### Indirect exchange of CTAC with citrate on the AuNT surface

The indirect exchange of CTAC with citrate was performed according to the modified literature procedure relying on the deposition and subsequent etching of an ultrathin Ag layer at the gold nanoparticle surface.^40^ The various initial volumes of the as-synthesized CTAC-capped AuNTs were centrifuged and re-dispersed in 2.0 mL of 0.1 mM CTAC. These initial volumes were adjusted in order to set similar total surface area values (Table S3:† resulting *S*_total_ varied insignificantly from 1.17 × 10^15^ to 1.29 × 10^15^ nm^2^), and hence, the required PVP amount (*m*_PVP_ varied from 1.166 to 1.285 mg). This was done to avoid the deviations in starting CTAC-AuNTs samples, which allowed keeping the amounts of PVA, AgNO_3_, and Na_3_Cit constant for each of the samples in the series.

The resulting 2.0 mL of CTAC-capped AuNTs were centrifuged one more time, in order to remove excessive surfactant molecules, and re-dispersed in 1 mL of 0.1 mM CTAC. The calculated amount of PVP (Table S3†) was dissolved in 3.8 mL of 0.1 mM CTAC and added to nanoparticle colloids together with 0.2 mL of 4% w/w PVA. To deposit an ultrathin Ag layer on the gold surface, 0.1 mL of 40 mM AA and 1.0 mL of 0.3 mM AgNO_3_ were injected subsequently. During shaking for 10 min the suspensions developed colors from blue to darker blue (with red scattering), violet, pink, and orange depending on the initial nanotriangle size (Fig. S6†). To remove CTAC, acetone was added at a 2 : 1 volume ratio (12.2 : 6.1 mL, to a total of 18.3 mL); the mixtures were shaken and centrifuged at lower g-forces than the initial CTAC-capped samples. The clear supernatants were discarded, and the precipitates were dried with a pressured nitrogen beam and re-dispersed in 1.0 mL of 0.1 mM Na_3_Cit. To etch silver, 0.9 mL of 3% H_2_O_2_ was added and the colloids were shaken for 3 h recovering the characteristic blue color in the first minutes of the reaction. The citrate-capped AuNTs were collected using centrifugation at lower g-forces than the initial samples, re-dispersed in 0.5 mL of 0.1 mM Na_3_Cit and incubated for 12 h at RT. The resulting colloids were mixed with 1.0 mL of ultrapure H_2_O, centrifuged and re-dispersed in 0.5 mL of ultrapure H_2_O.

#### Characterization techniques and instrumentation

The colloids of gold nanoparticles were characterized with ultraviolet-visible (UV–VIS) spectroscopy using a JASCO V-670 UV–VIS-NIR (Easton, PA, USA) spectrophotometer in a quarz glass SUPRASIL® precision cell (Hellma, Jena, Germany) with the following parameters: data interval – 1 nm, UV/Vis bandwidth – 1.0 nm, response – medium, scan speed – 400 nm min^−1^, light source – D2/WI, filter exchange – continuous, and correction – baseline.

Transmission electron microscopy (TEM) and scanning transmission electron microscopy (STEM) images were acquired using Hitachi HT7800 (Tokyo, Japan) and FEI Helios NanoLab G3 UC (Hillsboro, OR, USA) microscopes, respectively. 2.5 μL of the sample was deposited on a Formvar coated copper grid (Plano) and air-dried.

Zeta-potential and dynamic light scattering (DLS) measurements were performed with zetasizer ZEN3600 Malvern Instruments Ltd (Worcestershire, UK) in a disposable folded capillary cell (DTS 1070, 800 μl; Malvern Instruments Ltd, Worcestershire, UK) and disposable micro UV-cuvette (70 μL; BRAND GMBH + CO KG, Wertheim, Germany), respectively. The parameters for zeta-potential measurements were set as follows: material – Au spheres (RI: 0.3, absorption: 0.2), dispersant – water at a temperature of 25 °C (viscosity: 0.8872 cP, RI: 1.330, dielectric constant: 78.5), *F*(ka) selection – Smoluchowski model (*F*(ka) value: 1.50), equilibration time for temperature stabilization – 30 s, 3 measurements with no delay in between, and automatic measurement duration with at least 10 runs per measurement; the samples were approximately set in the range from 5.0 × 10^8^ to 1.0 × 10^10^ NP mL^−1^ in diluted ligand solutions. The DLS parameters: material – Au spheres (RI: 0.3, absorption: 0.2), dispersant – water at a temperature of 25 °C (viscosity: 0.8872 cP, RI: 1.330), measurement angle – 173° backscatter (NIBS default), equilibration time for temperature stabilization – 30 s, 3 measurements with no delay in between, and automatic measurement duration with at least 10 runs per measurement; the sample concentrations were approximately set in the range from 1.0 × 10^10^ to 8.0 × 10^10^ NP mL^−1^ in diluted ligand solutions.

The transmission Fourier-transform infrared (FTIR) spectra in the wavenumber range of 370 cm^−1^–8000 cm^−1^ were recorded in a vacuum (2 hPa) with a Bruker V80v spectrometer under the following conditions: SiC MIR source, 6 mm source aperture, KBr beam splitter, 10 kHz mirror speed, RT-DLaTGS detector, 3 mm sample opening, and 2 cm^−1^ spectral resolution. The KBr substrates freshly pressed in a clean room environment were used to drop-cast 20 μL of aqueous samples and air-dried for *ca.* 30 min. The concentrations were varied to achieve optimal absorption intensities (10 mM CTAC; 34 mM PSS; 100 mM Na_3_Cit; *ca.* 1.0 × 10^12^ NP mL^−1^ of CTAC-AuNTs in 10 mM CTAC; *ca.* 1.6 × 10^13^ NP mL^−1^ of PSS-AuNTs in 10 mM PSS; *ca.* 3.1 × 10^12^ NP mL^−1^ of citrate-AuNTs in 10 mM Na_3_Cit; the molarity of PSS here is based on the molecular weight of its monomeric unit *M*_m.u._ = 206.20 g mol^−1^).

The spectral data processing was performed using OriginLab software (OriginLab Corporation, Northampton, MA, USA). For clear visualization of the IR vibrational modes a baseline correction and an offset were applied to the FTIR spectra. The electron microscopy images were analyzed using ImageJ software (National Institutes of Health and University of Wisconsin, USA).

#### Evaluation of AuNT bulk sensitivity using the glucose test in solution

For bulk sensitivity measurements, CTAC-, PSS- and citrate-capped gold nanotriangles were centrifuged and re-dispersed in 0.1 mM CTAC, 0.01 mM PSS (based on its *M*_m.u._ = 206.20 g mol^−1^), and 0.1 mM Na_3_Cit, respectively. 26.67 μL of the concentrated colloids were mixed with 93.33 μL of 45% w/w d-glucose solution, which resulted in 120 μL of AuNTs in 35% w/w glucose with intensity sufficient for the UV–VIS measurements. The mixtures were then step-by-step diluted using ultrapure water to reach concentrations of glucose of 30, 25, 20, 15, and 10% w/w. After each addition and thorough mixing, the UV–VIS spectra were acquired (data interval: 0.2 nm, UV/Vis bandwidth: 1.0 nm, response: medium, scan speed: 200 nm min^−1^, light source: D2/WI, filter exchange: continuous, correction: baseline, 4 cycles, and cycle interval: 30 s). The 0% w/w corresponds to the initial samples in water without the addition of glucose. A digital refractometer PAL-RI (Atago, Tokyo, Japan) was used to measure the refractive index of the glucose solutions in the corresponding mixtures of ultrapure water and utilized ligands at room temperature (26–27 °C). Bulk sensitivity was determined by plotting the shift of the LSPR peak position as a function of the measured change in the refractive index.

## Results and discussion

**Scheme 1:**
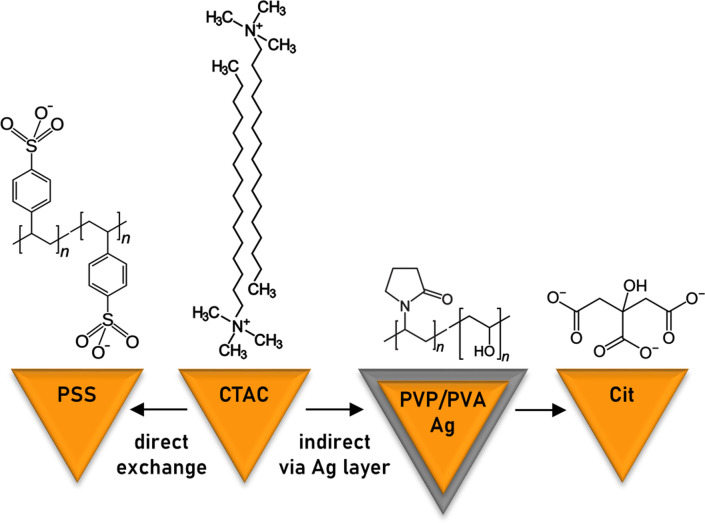
Studied pathways for ligand exchange on the surface of gold nanotriangles.

The two studied pathways for the ligand exchange on the surface of gold nanotriangles are depicted in Scheme 1. The direct replacement of cationic surfactant CTAC with anionic polyelectrolyte PSS is possible apparently due to the latter's bulky structure providing additional steric stabilization. Alternatively, it can be assumed that PSS forms a secondary layer on top of CTAC. In order to exchange CTAC with citrate, as a weaker ligand, we used the indirect method implementing the intermediate step of silver coating capped by a mixture of PVP and PVA. We show the calculation of gold nanotriangle surface area for estimating the required amount of the ligand, which allows adapting the procedure to each individual sample in a rational way. The correlations given below are helpful for estimating the required amount of the ligand in an express manner. However, for the most accurate calculations we recommend creating a calculator working with the initial constants and sample characteristics as an input.

### Calculation of the gold nanotriangle concentration and surface area

The average edge length of nanotriangles (*L*_NT_, nm) can be determined with statistical analysis of electron microscopy images or using an express method based on the LSPR peak position (*λ*_LSPR_, nm) in the UV–VIS spectrum. The latter utilizes the linear correlation function as was described earlier: *L*_NT_ = 0.6361 × *λ*_LSPR_ − 348.76.^53,54^ The thickness, (*T*_NT_, nm), of AuNTs is known to be relatively constant within the selected synthesis protocol and was considered to be *ca.* 26 nm for samples obtained with seed-mediated synthesis as reported by Szustakiewicz and co-workers.^53^ Knowing these values, one can easily calculate the average dimensions of a single gold nanotriangle, such as volume (*V*_NT_, nm^3^) and surface area (*S*_NT_, nm^2^), which are equal to √3/4 × *L*_NT_^2^ × *T*_NT_ and √3/2 × *L*_NT_^2^ + 3 × *T*_NT_ × *L*_NT_ for a triangular prism, respectively.

To estimate the concentration of gold nanotriangles (*C*_NT_, NP mL^−1^) we used the relation of the molar gold concentration (*C*_Au^0^_, mM) to absorbance at 400 nm (*A*_400_, a.u.). Based on LSPR simulations, UV–VIS analysis, and inductively coupled plasma mass spectrometry (ICP-MS) data, Scarabelli *et al.* showed that *A*_400_ = 1.2 corresponds to *C*_Au^0^_ = 0.5 mM regardless of the nanoparticle shape and size, which gives the ratio *C*_Au^0^_ (mM) = 0.42 × *A*_400_.^56,60^ This was recently validated by Khlebtsov and co-workers for a wide variety of other shapes including nanotriangles. The authors have derived the following correlation: *C*_Au^0^_ (mM) = 0.44 × *A*_400_.^61^ We established the current work before that publication and therefore kept the former equation in our calculations. The amount of gold per single nanotriangle is determined using its mass (*m*_NT_, g), and hence its volume, as in the following equation:1
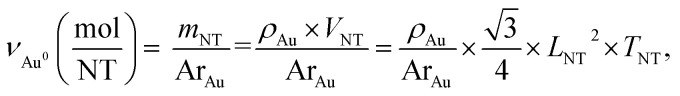
in which *ρ*_Au_ = 19.30 g cm^−3^ is the density of metallic gold and Ar_Au_ = 196.97 g mol^−1^ is its atomic weight. The gold nanotriangle concentration is a quotient of the total molar concentration of gold and its amount per single particle:2
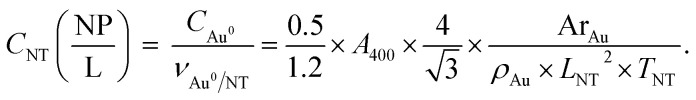


Taking into account all the constants and coefficients, we could obtain a simple correlation:3
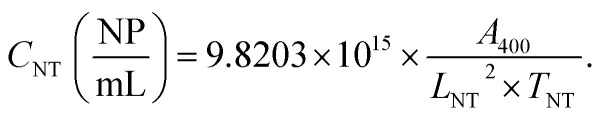


The total surface area of gold nanotriangles (*S*_total_, nm^2^) is the product of their concentration (*C*_NT_, NP mL^−1^), aliquot volume (*V*_total_, mL), and the surface area of a single nanoparticle (*S*_NT_, nm^2^):

4 *S*_total_ (nm^2^) = *C*_NT_ × *V*_al._ × *S*_NT_.

### Direct ligand exchange of CTAC with PSS

We first describe the direct exchange of CTAC with PSS on the gold nanotriangle surface. Its efficiency depends on binding competition between two ligands and, hence, requires an excessive amount of PSS together with incubation for a long period of time. Depending on the polymer chain length, the number of actual functional groups would vary. Therefore we prefer to consider the number of monomeric units (*n*, m.u.) rather than the number of molecules, which helps avoid misinterpretations and makes the calculations independent of the polymerization degree of the ligand in a specific batch. The preliminary tests were done to determine the required ratio of PSS monomeric units to the surface area of a nanotriangle. The value *R* = 10 800 m.u. nm^−2^ was found to provide the most reproducible results. Its product with total surface area of AuNTs in the sample returns the number of monomeric units sufficient to cover the nanoparticle surface:

5 *n*_PSS_ (m.u.) = *R* × *S*_total_.

The mass of PSS is calculated using the molar weight of its monomeric unit *M*_m.u._ (C_8_H_7_SO_3_Na) = 206.20 g mol^−1^ and the Avogadro number *N*_A_ = 6.022 × 10^23^:6
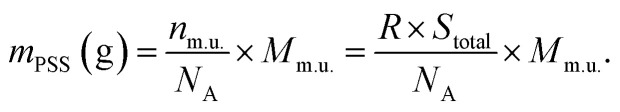


Taking into account all the constants and coefficients, the following correlation is obtained:

7 *m*_PSS_ (mg) = 3.6980 × 10^−15^ × *S*_total_.

**Fig. 1:**
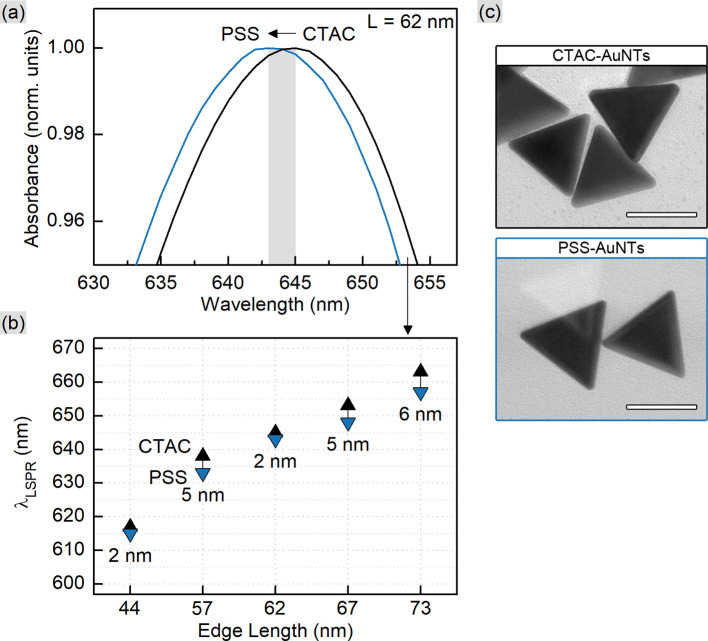
UV–VIS spectra (a) of CTAC- and PSS-capped 62 nm AuNTs. The peak positions (b) show the blue shift after the ligand exchange for the samples with various edge lengths (44, 57, 62, 67, and 73 nm as indicated by the *X*-axis). TEM micrographs of 62 nm AuNTs (c) reveal no morphology change after the process; scale bars are 50 nm.

This correlation together with eqn (3) and (4) allows rationally adapting the procedure to an individual sample with given AuNT dimensions and concentration. With this, the corresponding amount of PSS was mixed with gold nanotriangles of varied sizes (Table S1†). The TEM images reveal that the morphology of AuNTs is retained during the process, and no tip rounding is observed for PSS-capped samples (Fig. 1 and S1†). The acquired UV–VIS spectra demonstrate well-defined LSPR peaks in the range from 617 to 663 nm with a clear blue shift of 2–6 nm after the ligand exchange (Fig. 1). The full width at half maximum (FWHM) did not change significantly only having a minor increase by 1–6 nm for PSS-AuNTs (Fig. S2†). Recalculating the measurements to the initial sample volume showed only a minor loss of the absorption intensities indicating 50–85% yields in terms of nanoparticle concentration (Fig. S3 and Table S1†). These losses may be associated with the workflow during multiple centrifugation cycles followed by the discard of the supernatant.

**Fig. 2:**
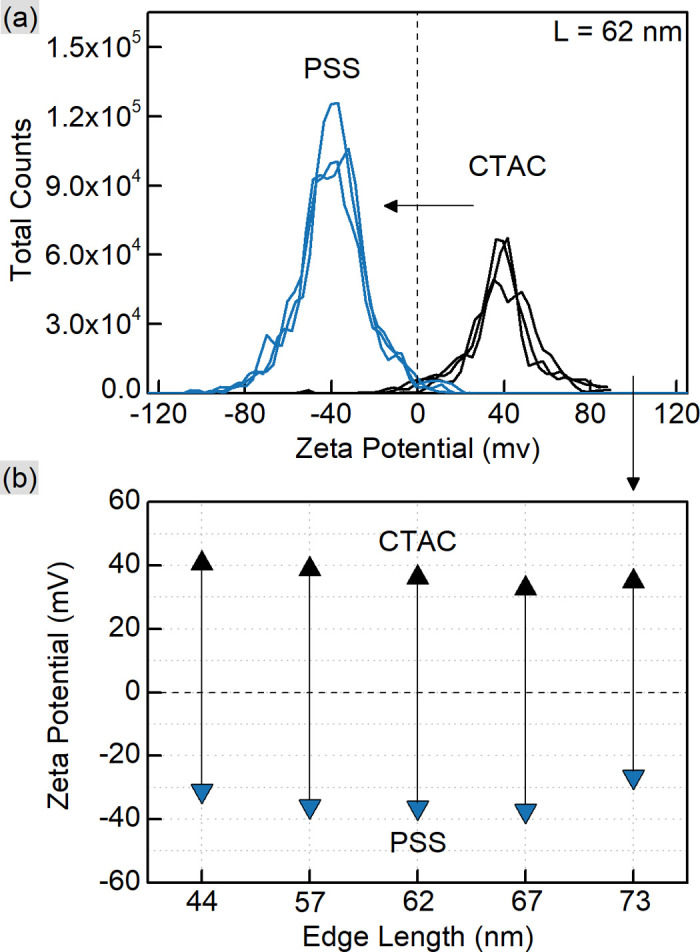
Zeta-potential measurements for CTAC- and PSS-capped AuNTs. The representative curves (a) are given for the 62 nm nanotriangles. The graph (b) shows the respective peak positions for AuNTs with various edge lengths (44, 57, 62, 67, and 73 nm as indicated by the *X*-axis).

CTAC is a quaternary amine known to form a bilayer on the gold nanoparticle surface, hence providing it with a positive charge.^30,32,33^ Exchanging CTAC with PSS, which has a negatively charged sulfonate group, leads to the corresponding zeta-potential change confirmed by the measurements (Fig. 2). Before and after the ligand exchange AuNTs demonstrate values from +41 to −37 mV, respectively, typical for well-stabilized colloids. The DLS measurements reveal a slight increase in hydrodynamic size after the ligand exchange which might be due to the bulkier structure of PSS as a polymer compared to the densely packed bilayer of CTAC. Alternatively, this may be caused by the formation of a secondary PSS layer on top of the existing CTAC bilayer.^62^ The supporting curves and numerical data are given in Fig. S4 and Table S2.†

**Fig. 3:**
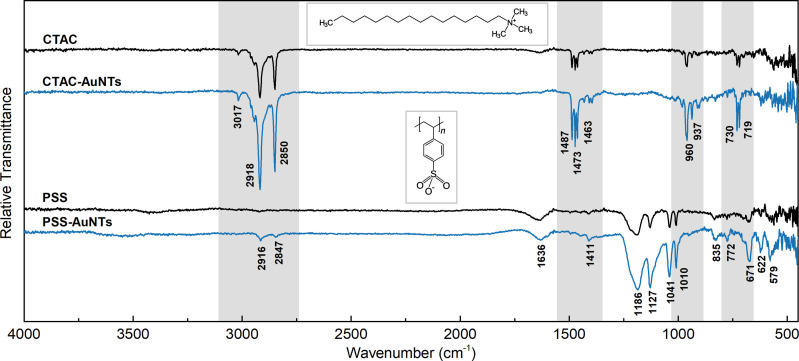
Relative vacuum transmittance FTIR spectra of CTAC- and PSS-capped nanotriangles (blue lines), and the reference spectra of pure ligands (black lines). The characteristic modes in the CTAC molecule are highlighted in grey.

To analyze the surface species, we recorded Fourier-transform infrared (FTIR) spectra of CTAC- and PSS-capped nanotriangles together with the reference data for pure ligands (Fig. 3). The alkyl tail of CTAC generates strong double peaks at 2918 and 2850 cm^−1^ due to anti-symmetrical and symmetrical –CH_2_– stretching.^63^ The bands at 1487, 1473, and 1463 cm^−1^ may be attributed to bending modes of methylene and methyl groups.^64^ The bands at 730 and 719 cm^−1^ are commonly assigned to –CH_2_– rock vibrations in relatively long hydrocarbon chains.^65,66^ The trimethylammonium head of CTAC provides a characteristic peak at 3017 cm^−1^ corresponding to the anti-symmetrical –CH_3_ stretching. The bands at 960 and 937 cm^−1^ are related to the C–N vibration of the cationic head of the cetrimonium ion.^65,66^ The same set of bands but of higher intensities appears in the spectrum of CTAC-capped AuNTs, which is due to the plasmonic enhancement.

The spectrum of PSS demonstrates the characteristic peaks for benzenesulfonic acids. The sulfonic acid group shows the strong stretching modes of S=O at 1186 cm^−1^ (anti-symmetrical) and 1041 cm^−1^ (symmetrical) together with lower intensity stretching vibrations of S–O at 622 and 579 cm^−1^.^67–70^ The C–H bending overtone at 1636 cm^−1^ is typical for aromatic compounds.^64,69,71^ The band at 1127 cm^−1^ corresponds to the in-plane skeleton vibration of the benzene ring.^67^ Additionally, the peaks at 1010 cm^−1^ and 671 cm^−1^ can be assigned to in-plane and out-of-plane bending skeleton vibrations.^67,68,71^ The bands at 1411, 835, and 772 cm^−1^ are generated by the C–H vibrational modes of the benzene group disubstituted in the *para* position.^64,67,72^ Similar to what was described above, the PSS-capped AuNTs retain the same peaks with an enhanced intensity. Additionally, the low-intensity stretching modes of the CH_2_ group were observed at 2916 and 2847 cm^−1^, which might be attributed to the residues of CTAC. This suggests that PSS forms a secondary coating layer without exchanging the surfactant. However, none of the other CTAC characteristic modes (at 1487–1463, 730–719 and 960–937 cm^−1^) are present in the curve allowing us to conclude that bands at 2916–2847 cm^−1^ might also arise due to the plasmonic enhancement of the signal from CH_2_ groups of PSS on the gold nanoparticle surface.

### Indirect ligand exchange of CTAC with citrate *via* the deposition and etching of an ultrathin silver layer

To cap nanoparticles with the relatively small and weakly binding anion of citrate, we applied the indirect exchange method. It involves the deposition of an ultrathin Ag layer in the presence of the PVP/PVA mixture, stabilizing the colloids against acetone. Once CTAC is removed from the mixture, the colloids are re-dispersed in water, and silver is etched with hydrogen peroxide in the presence of citrate. The ratio of 60 PVP molecules per nm^2^ was earlier reported by Graf and co-workers to be sufficient for covering various gold nanoparticles.^73^ However, the number of actual functional groups (monomeric units) would vary depending on the polymer chain length. For this reason we consider the degree of polymerization, which is 90 for PVP-10 having *M*_av._ = 10 000 g mol^−1^ and the molar weight of its monomeric unit *M*_m.u._ (C_6_H_9_NO) = 111.14 g mol^−1^. Therefore, the required ratio is *R* = 5400 m.u. nm^−2^. Similar to the calculations above (eqn (5) and (6)), the mass of PVP can be calculated using the molar weight of its monomeric unit and the Avogadro number. Taking into account all the constants and coefficients, the following correlation was obtained:

8 *m*_PVP_ (mg) = 9.9664 × 10^−16^ × *S*_total_.

To avoid significant deviation in the initial samples, we adjusted their concentrations setting similar total surface area values (and hence, the required PVP amount). The amount of PVA was fixed at 5 mg per sample giving the most reproducible results. The quantity of silver nitrate was kept the same as in the original procedure. The thickness of the Ag layer deposited onto the gold surface can be estimated based on the total surface area of AuNTs and the utilized amount of AgNO_3_ as described in the ESI.† For the studied samples, it was found to be *ca.* 2.5 nm (Table S3†), which fulfills the criteria of an Ag layer thicker than 1.5 nm for complete CTAC removal as reported by Zhou and co-authors.^40^

The amount of added ligand should be optimal not only in terms of the required minimum, but also not to be overabundant, as this may affect the long-term colloidal stability. With this in mind, we have estimated the necessary amount of citrate for each sample using the following equation:9
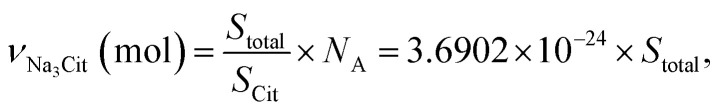
where *S*_Cit_ is the footprint of citrate on the Au (111) surface considered to be 0.45 nm^2^ per molecule.^74^ The values were found to be *ca.* 5 × 10^−9^ mol on average, which is 400-fold lower than the initially reported 2 × 10^−6^ mol (0.2 mL of 10 mM Na_3_Cit used in ref. 40). Introducing a lower 20-fold excess returns 1 × 10^−7^ mol, which is utilized in our modified procedure (1.0 mL of 0.1 mM Na_3_Cit). Lowering the citrate amount as described improved the yield due to lower aggregation of nanoparticles during the silver-etching step (Fig. S5†). Additionally, this provided better stability during centrifugation and long-term storage proving the need to critically adjust the ligand-to-nanoparticle ratios utilized in each procedure step.

**Fig. 4:**
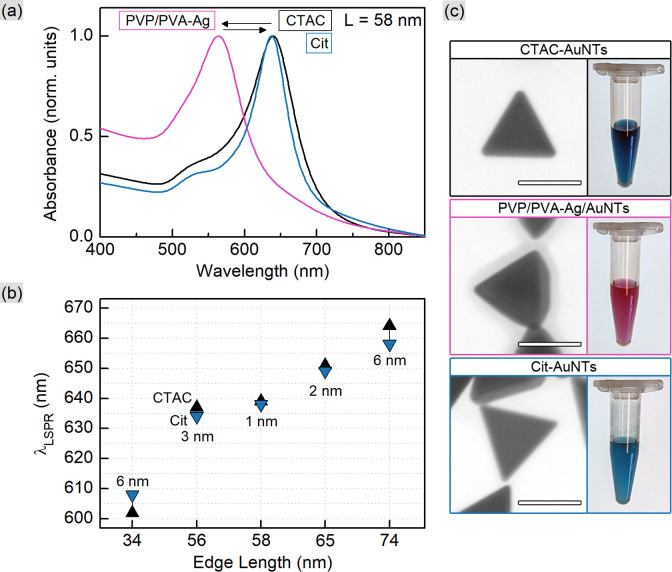
UV–VIS spectra (a) for 58 nm AuNTs during the indirect ligand exchange process. The LSPR peak positions (b) show the shift after the ligand exchange for the samples with various edge lengths (34, 56, 58, 65, and 74 nm as indicated by the *X*-axis). STEM micrographs of 58 nm AuNTs (c) reveal the morphology change at the intermediate step; scale bars are 50 nm.

Taking the above calculations into consideration, we prepared a series of citrate-capped AuNTs (Table S3†). Upon the silver layer deposition the colors of colloids developed from blue to darker blue, violet, pink, and orange depending on the nanotriangle size (Fig. S6†). The initial blue color was recovered after etching Ag with H_2_O_2_. The respective changes are vividly demonstrated by the blue-shift of the LSPR peak and its return nearly to the initial position (Fig. 4). The scanning transmission electron microscopy (STEM) images reveal the morphology to be slightly distorted for Ag-coated nanoparticles but to recover to the well-defined triangular shape after etching (Fig. 4 and S7†). The UV–VIS spectra of the resulting colloids show the retention of well-pronounced LSPR peaks in the range from 608 to 658 nm with a red shift of 1–6 nm after the ligand exchange, except for the 34 nm nanotriangles demonstrating a blue 6 nm shift. The FWHM of the peaks decreased by 12–24 nm which might be associated with losses caused by the use of acetone as an organic solvent and multiple steps in the procedure (Fig. S8†). The same reasoning has a final yield of 12–32% in terms of nanoparticle concentration (Fig. S9†), which is lower than that after the direct exchange of CTAC with PSS as described above. Noteworthily, the yield for such complex procedures of the indirect exchange frequently appears to be very poor or even remains unspecified.

**Fig. 5:**
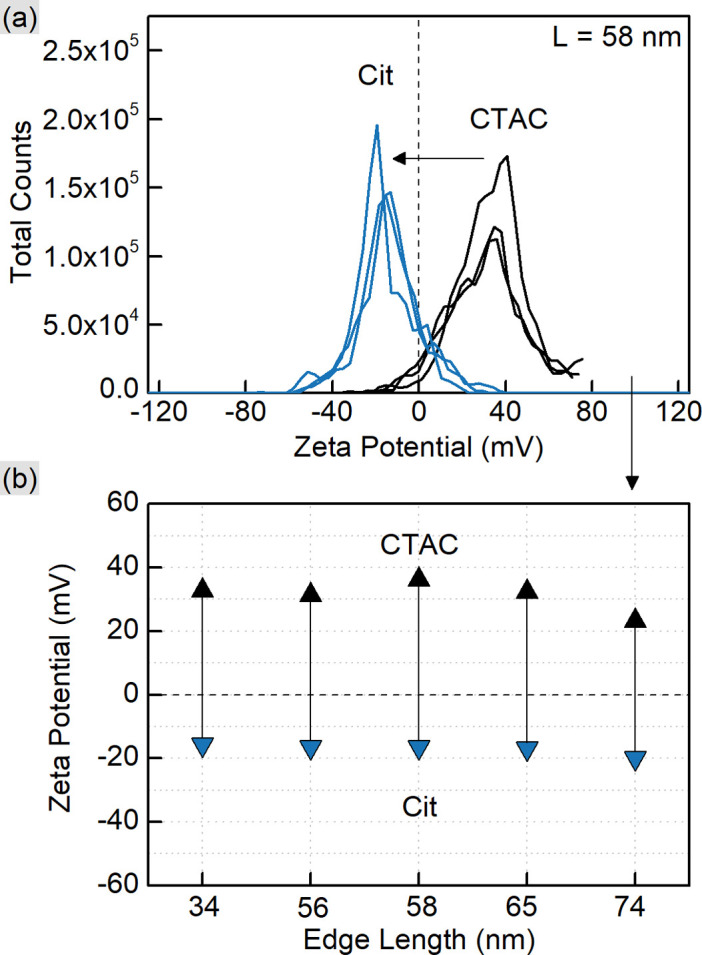
Zeta-potential measurements for CTAC- and citrate-capped AuNTs. The representative curves (a) are given for the 58 nm nanotriangles. The graph (b) shows the respective peak positions for AuNTs with various edge lengths (34, 56, 58, 65, and 74 nm as indicated by the *X*-axis).

As shown earlier, CTAC-capped gold nanotriangles have a positive zeta-potential, which was determined in the range from +33 up to +41 mV for the studied samples (Fig. 5 and S10, Table S4†). Upon Ag deposition, the core–shell structures covered with the mixture of PVP and PVA typically show a zeta potential of *ca.* −13 mV. After silver etching and introducing citrate onto the nanoparticle surface, they acquired a stronger negative charge of −16 and up to −20 mV indicating relatively stable colloids. DLS showed a significant increase in hydrodynamic size, which might be due to the lower electrostatic repulsion and steric stabilization compared to the previously used surfactant or polymer molecules (Fig. S10 and Table S4†).

**Fig. 6:**
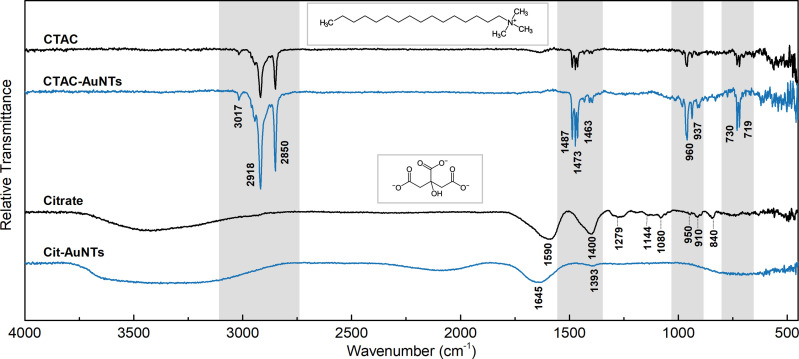
Relative vacuum transmittance FTIR spectra of CTAC- and citrate-capped nanotriangles (blue lines), and the reference spectra of the pure ligands (black lines). The characteristic modes in the CTAC molecule are highlighted in grey.

The acquired FTIR spectrum of citrate (Fig. 6) is in agreement with earlier published data for its fingerprint area.^75^ The broad band at *ca.* 3400 cm^−1^ corresponds to O–H stretching.^64^ The peaks at 1590 and 1400 cm^−1^ are generated by anti-symmetrical and symmetrical stretching of carboxylic groups.^76^ The low-intensity bands in the region of 1279–1080 cm^−1^ are due to the C–O stretching modes, while the one at 840 cm^−1^ is affiliated to the carboxylic group bending.^76^ The peaks at 950–910 cm^−1^ may be associated with O–H bending.^63^ Upon binding to the nanoparticle surface, most of the vibrational peaks weaken and disappear indicating the rigid state of the citrate molecule. The anti-symmetrical CO_2_^−^ stretching band shifts to 1645 cm^−1^ while the symmetrical stretching mode significantly decreases in intensity with a minor low-frequency shift to 1393 cm^−1^ as was observed earlier by Wulandari and co-workers.^76^ None of the characteristic peaks for CTAC (see the section above) are found in the spectrum of citrate-capped AuNTs confirming rather complete removal of surfactant molecules from the nanoparticle surface.

### Bulk refractive index sensitivity of gold nanotriangles

**Fig. 7:**
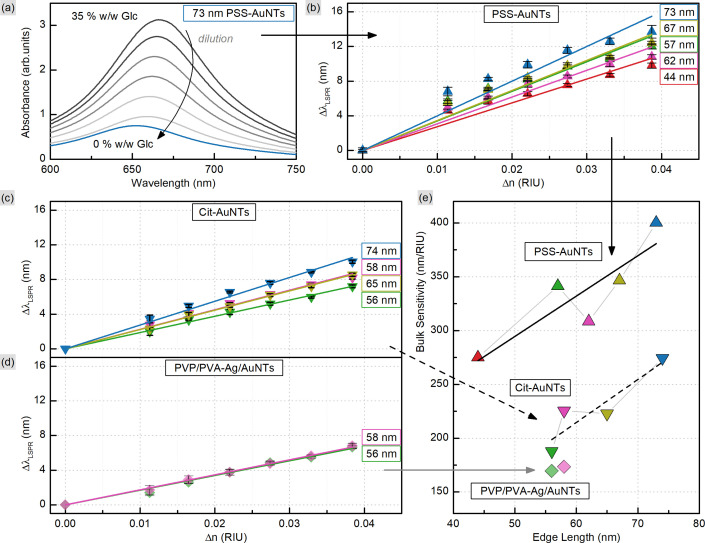
Determination of bulk sensitivity (e) based on the plasmonic shift (Δ*λ*_LSPR_) as a response to the change in the refractive index of the medium (Δ*n*) upon glucose addition (a) to AuNTs with various dimensions and surface ligands (b–d).

In order to evaluate the sensitivity of manufactured nanotriangles we carried out a simple test by tuning the refractive index of the solution. The latter was done by varying the glucose (Glc) concentration from 35 to 10% w/w with step-by-step dilution, while the 0% w/w corresponds to the initial samples in water. The CTAC-capped AuNTs did not withstand the glucose addition to a final concentration of 30% w/w: their colloids turned colorless losing the LSPR peak in the UV–VIS spectrum (Fig. S12†). Nevertheless, functionalizing nanotriangles with PSS or citrate allowed for retaining the characteristic band and measuring its response. As the refractive index increased from *n*_0%_ = 1.332 to *n*_35%_ = 1.371 RIU (refractive index units), the LSPR peaks demonstrated red-shifts for each of the samples (Fig. S13–S15†). Plotting the resonance shift (Δ*λ*_LSPR_, nm) against the change in the refractive index of the medium (Δ*n*, RIU) provides a graph to determine bulk refractive index sensitivity (*S*_B_, nm/RIU). It is commonly defined as the ratio of the mentioned values (*S*_B_ = Δ*λ*_LSPR_/Δ*n*) and hence can be calculated as the slope parameter of a linear fit function (Fig. 7). This characteristic is known to depend on nanoparticle properties such as their material, size and shape.^59^ Larger nanoparticles are typically characterized by higher bulk refractive index sensitivity, which explains its rise with the increase in nanotriangle edge length in our study (Fig. 7). Interestingly, 57 nm PSS-AuNTs and 58 nm citrate-AuNTs demonstrate relatively increased *S*_B_ values breaking the linear trend in this dependency.

**Fig. 8:**
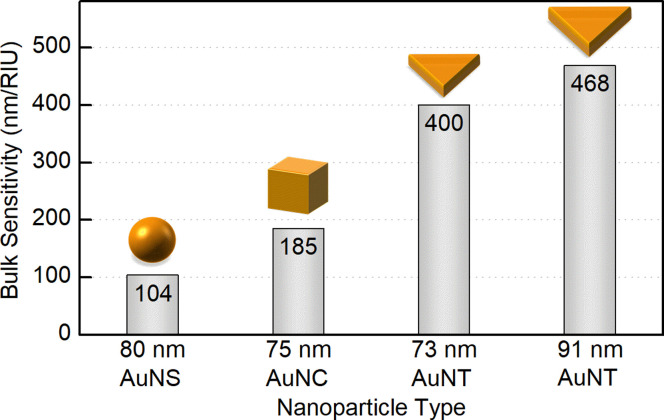
Bulk refractive index sensitivity depending on the shape and dimensions of gold nanoparticles.

The highest *S*_B_ = 400 nm/RIU was detected for 73 nm PSS-capped AuNTs (Table S5†). Nanotriangles of this size are the largest among those tested in this study, which determines their highest sensitivity. This value is 4-fold higher than the sensitivity earlier determined with the same method for 80 nm gold nanospheres (AuNSs, *S*_B_ = 104 nm/RIU)^77^ and 2-fold higher than the one of 75 nm gold nanocubes (AuNCs, *S*_B_ = 185 nm/RIU).^78^ This demonstrates a great potential for utilizing nanotriangles in LSPR-based sensing compared to the majority of morphologies studied earlier (Fig. 8).^9^ Additionally, the obtained data agree with the sensitivity values reported before for photocatalytically derived AuNTs, which demonstrated slightly higher sensitivity (*S*_B_ = 468 nm/RIU) likely due to their larger sizes (edge length was *ca.* 91 ± 17 nm).^8^

**Fig. 9:**
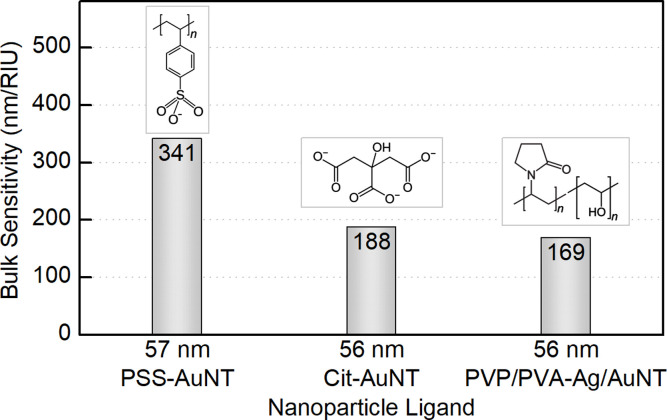
Bulk refractive index sensitivity depending on the surface ligands of gold nanotriangles.

Nevertheless, when comparing sensitivity values one should keep in mind that it highly depends not only on internal nanoparticle properties, but can also be affected by other factors, such as surface ligands. For PSS-capped AuNTs, bulk sensitivity was found to vary from 274 to 400 nm/RIU depending on nanotriangle size (Table S5†). Meanwhile citrate-capped AuNTs with comparable dimensions demonstrate significantly, *ca.* 1.5-fold, lower sensitivity values in the range from 187 to 274 nm/RIU (Table S6†). And finally the intermediate products of the indirect ligand exchange, Ag-coated AuNTs capped with the mixture of PVP/PVA, have the lowest sensitivity detected in this series: from 170 to 173 nm/RIU. The observed influence of ligands on bulk refractive index sensitivity (Fig. 9) is apparently caused by the differences in their physical and chemical properties. Variation in these characteristics changes the medium in the nearest vicinity of the nanoparticle surface as well as the character of its interaction with functionalizing and analyte molecules. Altogether this highlights that any reference values from distinct sources should be compared carefully taking into account every difference in the studied systems and experiment design.

## Conclusions

We describe a rational estimation of the ligand amount required for the surface functionalization of gold nanotriangles based on their dimensions and concentration, which are obtained from express UV–VIS analysis. Determining the total surface area as a function of both characteristics provides excellent versatility for numerous protocols allowing adapting them for every individual sample (*e.g.* for AuNTs with varied edge lengths from 30 to 80 nm). Based on multiple characterization techniques it is shown to effectively work in exchange of CTAC with PSS with the direct method and with citrate *via* the deposition and subsequent etching of a silver layer (*ca.* 2.5 nm thick). While a relatively simple direct exchange provides a high yield of negatively charged PSS-capped AuNTs, the indirect approach provides a reproducible method for complete exchange of a densely packed surfactant with the weaker binding citrate. Moreover, this approach has the potential for switching to other morphologies by only changing the geometry formulae for the volume and surface area of a single nanoparticle.

The exchange of CTAC in both ways enabled evaluating the bulk sensitivity of the prepared AuNTs. The strongest response to the change in the refractive index of the medium was shown by 73 nm PSS-capped gold nanotriangles, which also demonstrated a significant increase compared to the 80 nm nanospheres and 75 nm nanocubes studied earlier. The citrate-capped AuNTs showed *ca.* 1.5-fold lower values proving that the bulk sensitivity highly depends not only on the internal nanoparticle properties, but also on external factors such as surface coating, and hence is highly affected by the experiment design. Nevertheless, nanoparticles capped with citrate often appear advantageous thanks to their biocompatibility and excellent application versatility.

## Data availability

The data supporting this article have been included as part of the ESI.†

## Author contributions

Conceptualization – E. P., A. C., and W. F.; methodology and investigation – E. P., S. E. S., and B. C.; data curation – E. P. and S. E. S.; formal analysis – E. P.; visualization – E. P.; writing – original draft – preparation – E. P.; writing – review & editing – A. C. and W. F.; supervision – A. C., W. F., and G. Z.; funding acquisition – A. C., W. F., and G. Z.; project administration – A. C., W. F., and G. Z. All authors have read and agreed to the published version of the manuscript.

## Conflicts of interest

There are no conflicts to declare.

## Supplementary Material

NA-OLF-D4NA00352G-s001
