# Supplementary material for: Customizable ligand exchange on the surface of gold nanotriangles enables their application in LSPR-based sensing
Source: Nanoscale Adv. 2024 Sep 5;6(21):5430–40. doi: 10.1039/d4na00352g (PMC11375502; doi:10.1039/d4na00352g)
Supplement: NA-006-D4NA00352G-s001 [file NA-006-D4NA00352G-s001.pdf]

Supplementary Information (SI) for Nanoscale Advances.
This journal is © The Royal Society of Chemistry 2024

Electronic Supplementary Information

Customizable ligand exchange on the surface of gold nanotriangles enables their application in LSPR-based sensing

Ekaterina Podlesnaia,[*a] Sarmiza Elena Stanca,[b] Buşra Çinçin,[a] Gabriel Zieger,[b] Andrea Csáki [a] and Wolfgang Fritzsche [*a]

a. Department of Nanobiophotonics, Leibniz Institute of Photonic Technology (Leibniz-IPHT), Member of the Leibniz Research Alliance – Leibniz Health Technologies, Albert-Einstein-Straße 9, 07745 Jena, Germany.

b. Quantum Detection Department, Leibniz Institute of Photonic Technology (Leibniz-IPHT), Member of the Leibniz Research Alliance – Leibniz Health Technologies, Albert-Einstein-Straße 9, 07745 Jena, Germany.

Table S1. Spectral characteristics of CTAC- and PSS-capped gold nanotriangles. V_{IS} indicates the volume of intermediate seeds used during the synthesis to obtain nanotriangles of various sizes. The estimation of PSS amount is done for 3.8 mL of as-synthesized CTAC-AuNTs. The absorbance at 400 nm for PSS-AuNTs was recalculated according to the initial sample volume in order to compare the concentrations and hence calculate the yield. For more details, see the main text.

CTAC-AuNTs	V_{IS}, µL	300	100	80	60	40
	λ_{LSPR}, nm	617	638	645	653	663
	FWHM, nm	147	103	111	85	92
	**L_{NT}, nm**	**44**	**57**	**62**	**67**	**73**
	A_{400}, a.u.	0.53053	0.98787	1.24216	0.6658	0.69931
	C_{NT}, NP/mL	1.05E+11	1.14E+11	1.24E+11	5.66E+10	4.96E+10
	S_{total}, nm^2	2.02E+15	3.17E+15	3.80E+15	1.95E+15	1.94E+15
	m_{PSS}, mg	7.459	11.701	14.062	7.195	7.179
PSS-AuNTs	λ_{LSPR}, nm	615	633	643	648	657
	$\Delta\lambda_{LSPR}$, nm	2	5	2	5	6
	FWHM, nm	-	109	113	88	93
	A_{400}, a.u.	0.31751	0.8369	0.78612	0.45975	0.35267
	C_{NT}, NP/mL	6.27E+10	9.70E+10	7.84E+10	3.91E+10	2.50E+10
	**Yield, %**	**60**	**85**	**63**	**69**	**50**

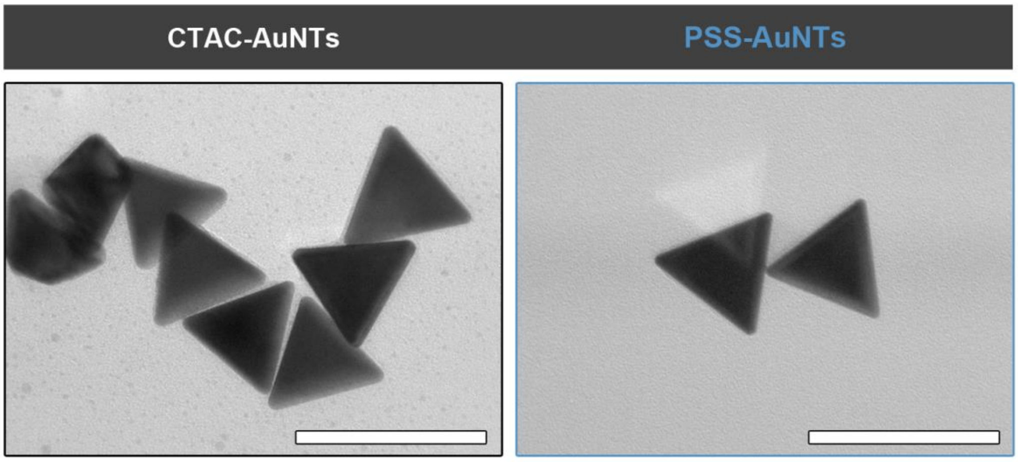

Figure S1. TEM images of 62 nm CTAC- and PSS-capped AuNTs, before and after direct ligand exchange, respectively. The scale bars are 100 nm.

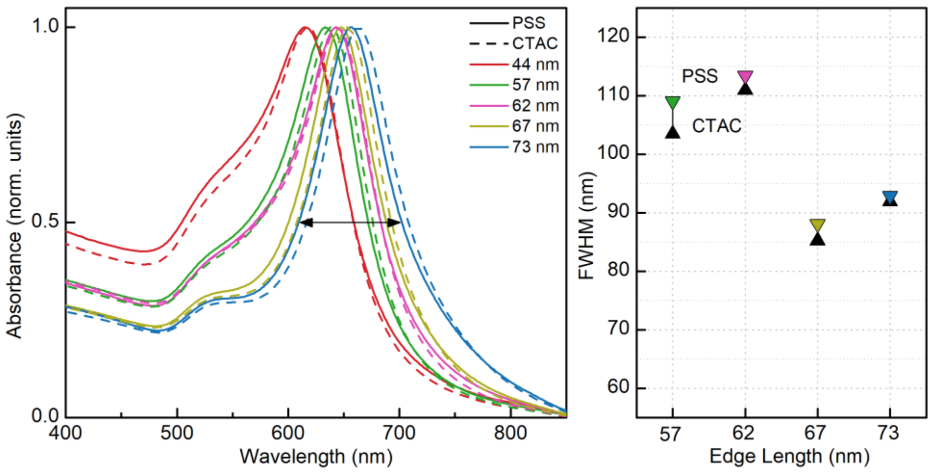

Figure S2. Normalized UV–VIS spectra (left) of CTAC- (dashed lines) and PSS-capped (solid lines) gold nanotriangles with various edge lengths (44, 57, 62, 67, and 73 nm as indicated in the legend) showing the blue shift. The FWHM of the peaks (right) were calculated using the integration tool in OriginLab.

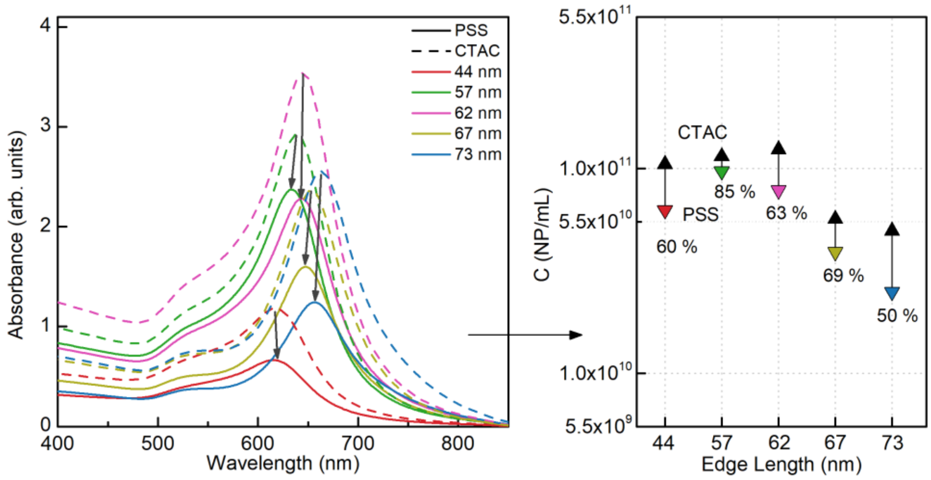

Figure S3. UV–VIS spectra (left) of CTAC- (dashed lines) and PSS-capped (solid lines) gold nanotriangles with various edge lengths (44, 57, 62, 67, and 73 nm as indicated in the legend). The curve intensities were recalculated to the initial sample volume in order to compare the concentrations (right) before (light gray) and after (dark gray) the direct ligand exchange. The percentages indicate the sample recovery yields in terms of nanoparticle concentration.

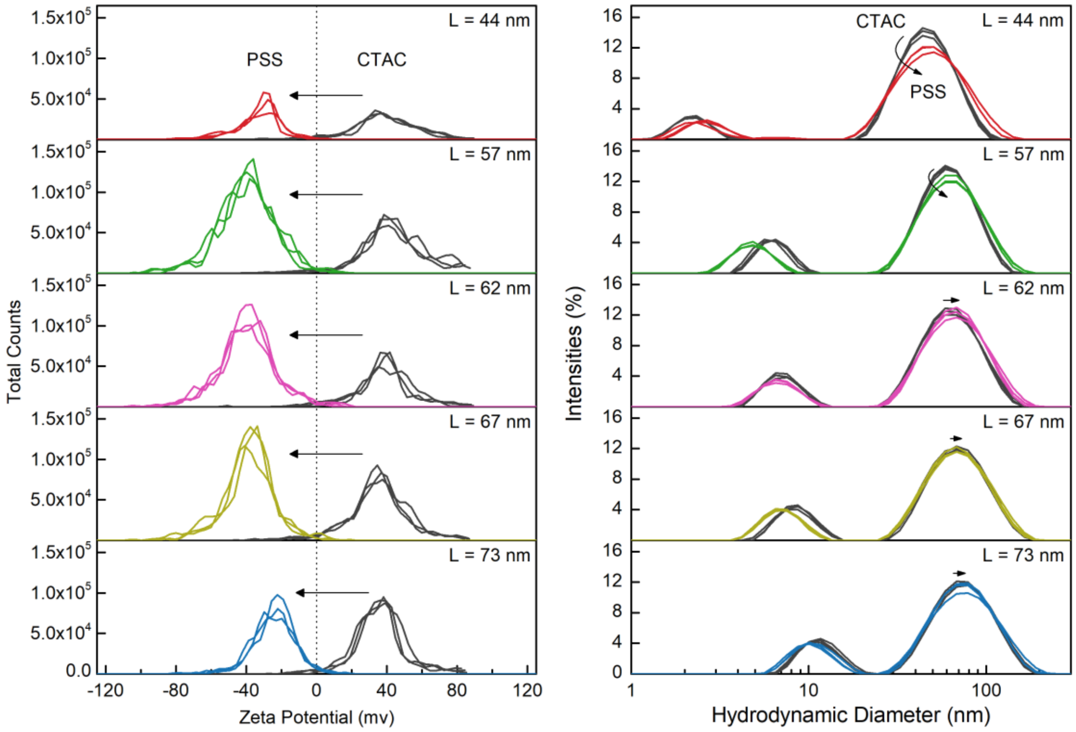

Figure S4. Zeta-potential (left) and DLS (right) curves for CTAC- (dark grey lines) and PSS-capped (colored lines) gold nanotriangles with various edge lengths (44, 57, 62, 67, and 73 nm).

Table S2. Zeta-potentials and hydrodynamic diameters of gold nanotriangles before and after direct exchange of CTAC to PSS.

Nanotriangle Edge Length, nm	Zeta-Potential, mV		Hydrodynamic Diameter, nm	
	CTAC-AuNTs	PSS-AuNTs	CTAC-AuNTs	PSS-AuNTs
44	+ 41	– 31	48.5	52.0
57	+ 39	– 36	63.9	67.7
62	+ 36	– 36	67.0	71.5
67	+ 33	– 37	72.9	73.2
73	+ 35	– 27	79.2	79.8

Table S3. Spectral characteristics of CTAC- and citrate-capped gold nanotriangles. V_{IS} indicates the volume of intermediate seeds used during the synthesis to obtain nanotriangles of various sizes. The estimation of PVP amount was done for 2.0 mL of utilized CTAC-AuNTs (as an alternative, a stock of 1.0 mg/mL PVP can be used, the required number of grams is then equal to the number of milliliters to add; the final added volume should be adjusted with 0.1 mM CTAC to 3.8 mL). The absorbance at 400 nm for citrate-AuNTs was recalculated according to the initial sample volume in order to compare the concentrations and hence calculate the yield. For more details, see the main text.

CTAC-AuNTs	V_{IS}, µL	300	100	80	60	40
	λ_{LSPR}, nm	602	637	639	651	664
	FWHM, nm	131	97	92	81	92
	**L_{NT}, nm**	**34**	**56**	**58**	**65**	**74**
	A_{400}, a.u.	0.542325	0.689184	0.726354	0.75364	0.831427
	C_{NT}, NP/mL	1.76E+11	8.16E+10	8.16E+10	6.66E+10	5.80E+10
	S_{total}, nm^2	1.29E+15	1.17E+15	1.21E+15	1.17E+15	1.21E+15
	T_{Ag}, nm	2.39	2.64	2.55	2.63	2.55
	m_{PVP}, mg	1.285	1.166	1.208	1.168	1.205
citrate-AuNTs	λ_{LSPR}, nm	608	635	638	649	658
	$\Delta\lambda_{LSPR}$, nm	-6	2	1	2	6
	FWHM, nm	-	73	68	69	77
	A_{400}, a.u.	0.08364	0.22744	0.38371	0.16908	0.16536
	C_{NT}, NP/mL	2.70E+10	2.70E+10	4.30E+10	1.50E+10	1.15E+10
	**Yield, %**	**12**	**20**	**32**	**23**	**23**

The thickness of a silver layer deposited onto the AuNT surface is calculated as the quotient of Ag volume and the total nanoparticle surface area:

$$T_{Ag} = \frac{V_{Ag}}{S_{total}}\ (\text{nm}).$$

The utilized amount of Ag was kept the same for each sample by adjusting the initial AuNT concentration to set the S_{total} having comparable values $(1.17 \times 10^{15} - 1.29 \times 10^{15} \text{nm}^2)$. The mass of silver ($m_{Ag}$) is the product of its atomic weight $(Ar_{Ag} = 107.8682\ \text{g/mol})$, silver nitrate concentration $(C_{AgNO_3} = 0.3\ \text{mM})$ and its volume $(V_{AgNO_3} = 1\ \text{mL})$:

$$m_{Ag} = Ar_{Ag} \times \nu_{Ag} = Ar_{Ag} \times C_{AgNO_3} \times V_{AgNO_3}.$$

The volume of silver is the quotient of its mass and density $(\rho_{Ag} = 10.49\ \text{g/cm}^3)$, which can be calculated as follows:

$$V_{Ag} = \frac{m_{Ag}}{\rho_{Ag}} = \frac{0.3\times10^{-3}\times1\times10^{-3}\times107.8682}{10.49} \times 10^{21} = 3.08 \times 10^{15}\ (\text{nm}^3).$$

Dividing this value by the total AuNT surface area for every individual sample returns the thickness values in the range from 2.39 to 2.64 nm (Table S3), which fulfills the criteria of Ag layer to be thicker than 1.5 nm for a complete CTAC removal as reported by Zhou and co-authors. [40]

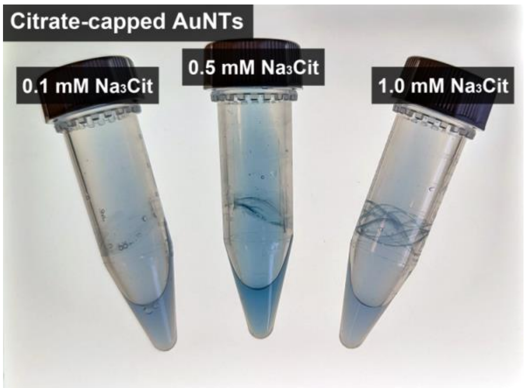

Figure S5. The citrate-capped AuNTs after the silver etching step. The colloids show different degree of agglomeration on tube walls depending on the concentration of Na_3Cit (1.0 mL) they were re-dispersed in.

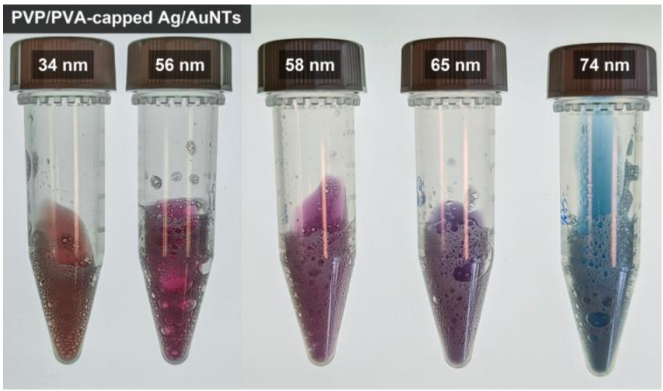

Figure S6. The colors of PVP/PVA-capped AuNTs covered with an ultrathin layer of silver depending on their initial size (edge length as indicated by the labels) during the indirect ligand exchange from CTAC to citrate.

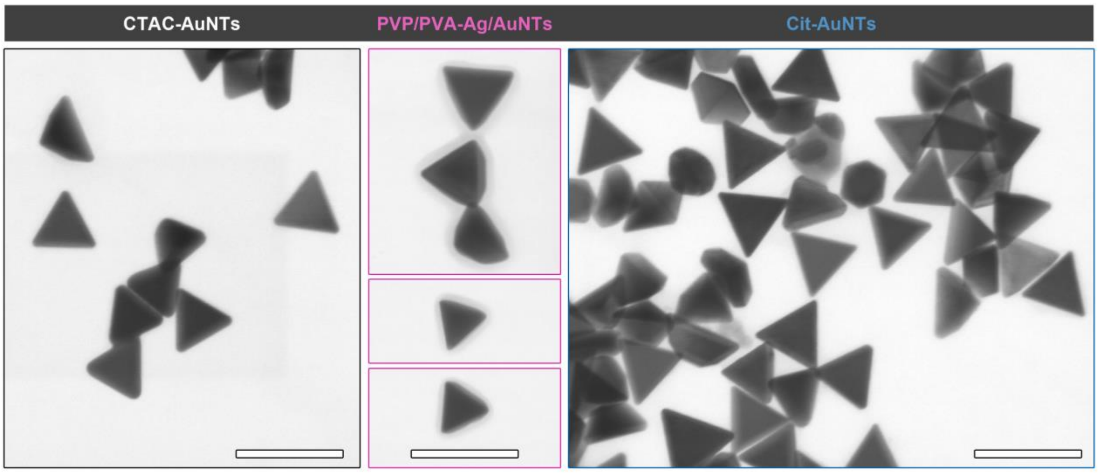

Figure S7. STEM images of 58 nm AuNTs during indirect ligand exchange process. The scale bars are 100 nm.

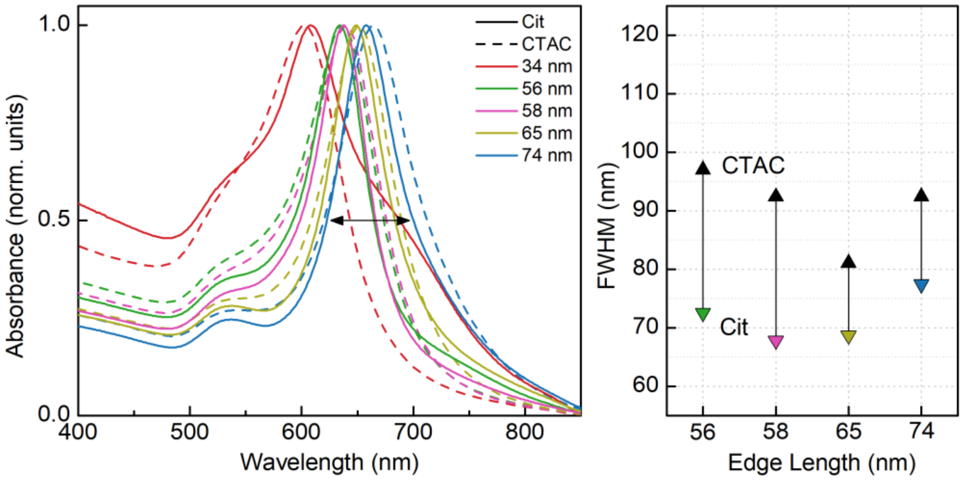

Figure S8. Normalized UV–VIS spectra (left) of CTAC- (dashed lines) and citrate-capped (solid lines) gold nanotriangles with various edge lengths (34, 56, 58, 65, and 74 nm as indicated in the legend) showing the blue shift except for the 34 nm AuNTs. The FWHM of the peaks (right) were calculated using the integration tool in OriginLab.

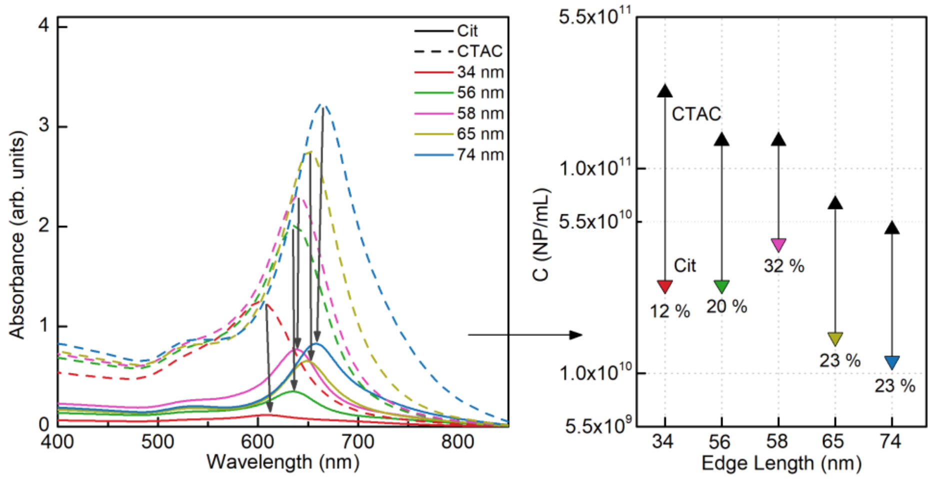

Figure S9. UV–VIS spectra (left) of CTAC- (dashed lines) and citrate-capped (solid lines) gold nanotriangles with various edge lengths (34, 56, 58, 65, and 74 nm as indicated in the legend). The curve intensities were recalculated to the initial sample volume in order to compare the concentrations (right) before (light gray) and after (dark gray) the indirect ligand exchange. The percentages indicate the sample recovery yields in terms of nanoparticle concentration.

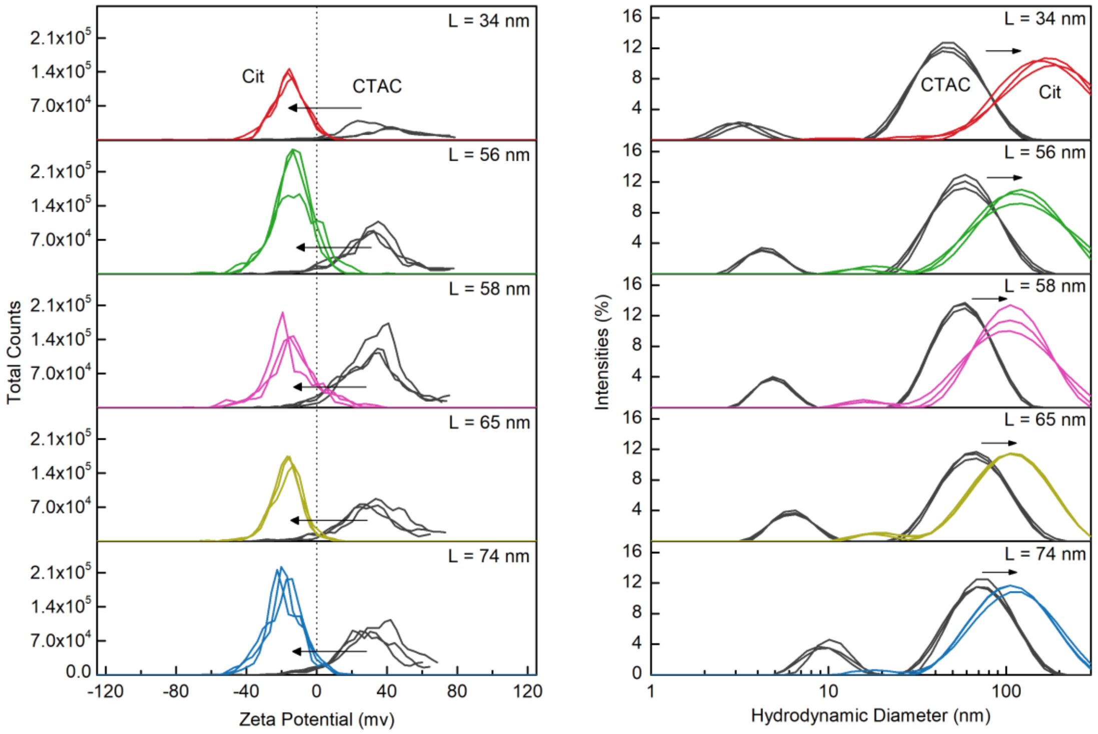

Figure S10. Zeta-potential (left) and DLS (right) curves for CTAC- (dark grey lines) and citrate-capped (colored lines) gold nanotriangles with various edge lengths (34, 56, 58, 65, and 74 nm).

Table S4. Zeta-potentials and hydrodynamic diameters of gold nanotriangles before and after indirect exchange of CTAC to citrate.

Nanotriangle Edge Length, nm	Zeta-Potential, mV		Hydrodynamic Diameter, nm	
	CTAC-AuNTs	Cit-AuNTs	CTAC-AuNTs	Cit-AuNTs
34	+ 33	– 16	50.7	190.2
56	+ 31	– 17	62.9	136.8
58	+ 36	– 16	60.3	115.5
65	+ 32	– 17	71.1	116.3
74	+ 23	– 20	77.3	119.4

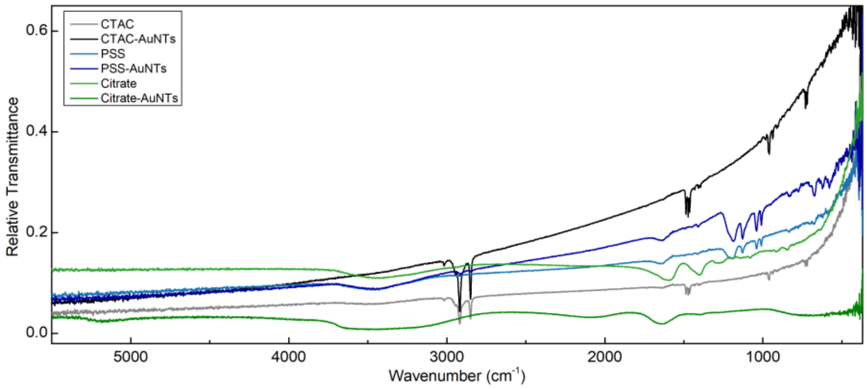

Figure S11. Raw FTIR spectra of CTAC-, PSS-, and citrate-capped AuNTs and pure ligands.

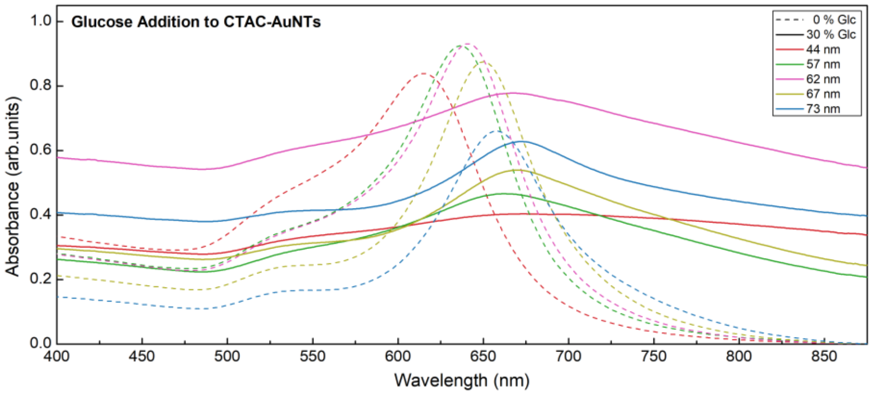

Figure S12. UV–VIS spectra of CTAC-capped gold nanotriangles with various edge lengths (L = 44, 57, 62, 67 and 73 nm as indicated in the legend) in 0 (dashed lines) and 30% w/w (solid lines) glucose concentrations showing the aggregation (LSPR band disappears) during the experiment.

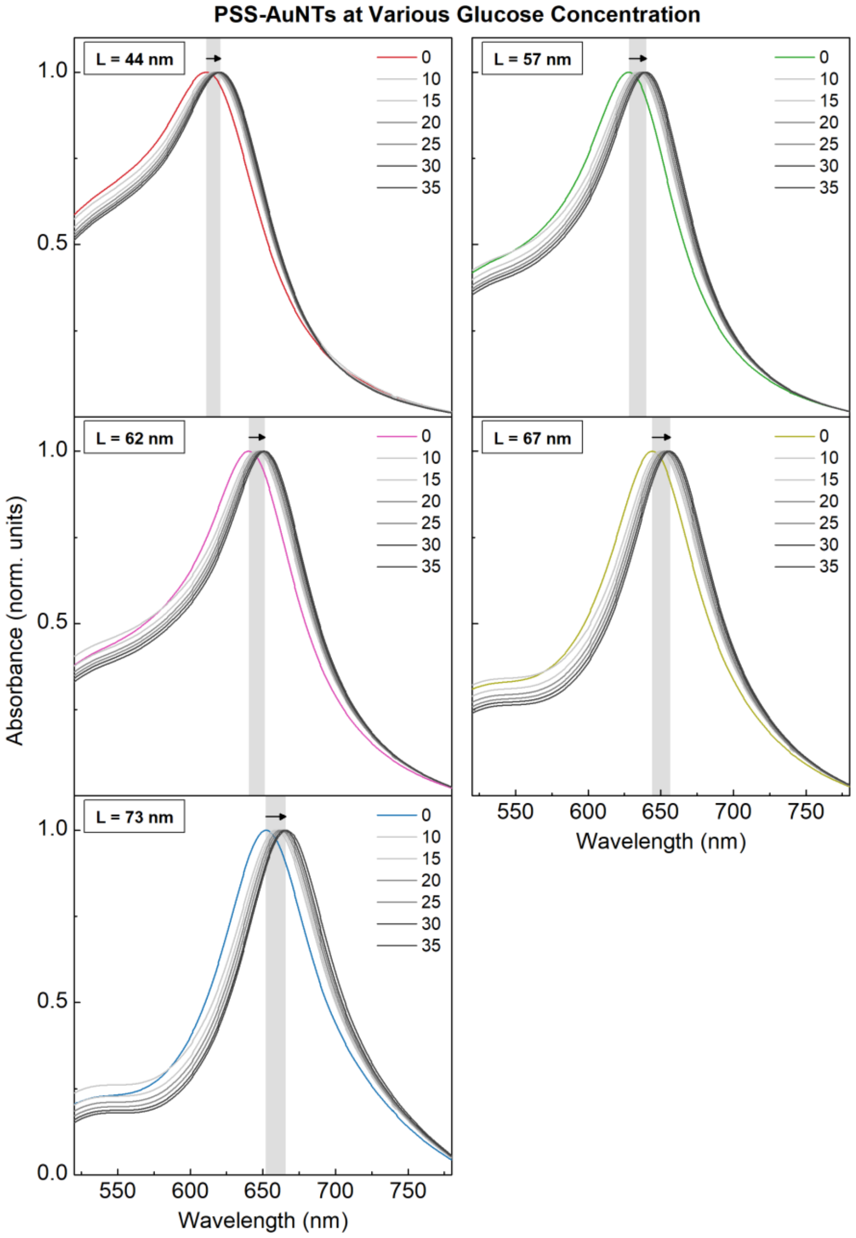

Figure S13. Normalized UV–VIS spectra of PSS-capped gold nanotriangles with various edge lengths (L = 44, 57, 62, 67 and 73 nm as indicated in the graph titles) at different glucose concentrations (in % w/w as indicated in legends). The plasmonic shifts are highlighted with arrows and grey areas upon the increase of glucose content.

Table S5. Determination of the bulk sensitivity (S_B) for PSS-AuNTs with various edge lengths. The S_B and adj. R^2 values were calculated with a linear fit function in OriginLab software.

C (Glu), % w/w	RI, RIU	mean λ_{LSPR} ± st.dev., nm				
		**44 nm PSS-AuNTs**	**57 nm PSS-AuNTs**	**62 nm PSS-AuNTs**	**67 nm PSS-AuNTs**	**73 nm PSS-AuNTs**
0	1.3321	611.1 ± 0.3	628.1 ± 0.3	640.5 ± 0.1	644.1 ± 0.1	652.2 ± 0.0
10	1.3435	615.7 ± 0.2	633.8 ± 0.3	645.4 ± 0.0	649.6 ± 0.3	659.0 ± 0.5
15	1.3489	616.8 ± 0.3	635.2 ± 0.2	646.6 ± 0.2	651.1 ± 0.1	660.5 ± 0.2
20	1.3542	617.6 ± 0.2	636.3 ± 0.1	648.1 ± 0.4	652.5 ± 0.1	662.1 ± 0.4
25	1.3595	618.6 ± 0.2	637.8 ± 0.2	649.0 ± 0.2	653.9 ± 0.3	663.8 ± 0.3
30	1.3652	619.8 ± 0.2	638.7 ± 0.1	650.4 ± 0.3	654.9 ± 0.2	664.8 ± 0.4
35	1.3708	620.9 ± 0.3	640.1 ± 0.3	651.3 ± 0.3	656.5 ± 0.2	665.9 ± 0.7
**S_B, nm/RIU:**		**275.0**	**341.4**	**308.5**	**346.6**	**400.2**
adj. R^2		0.98419	0.98209	0.98584	0.98668	0.97909

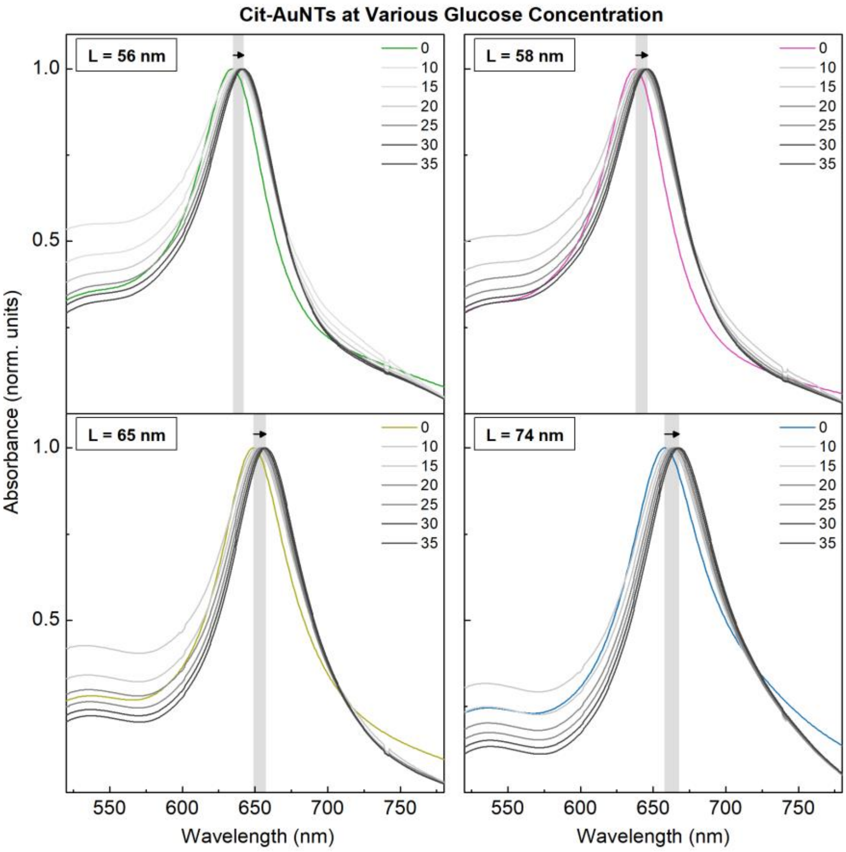

Figure S14. Normalized UV–VIS spectra of citrate-capped gold nanotriangles with various edge lengths (L = 34, 56, 58, 65, and 74 nm as indicated in the graph titles) at different glucose concentrations (in % w/w as indicated in legends). The plasmonic shifts are highlighted with arrows and grey areas upon the increase of glucose content.

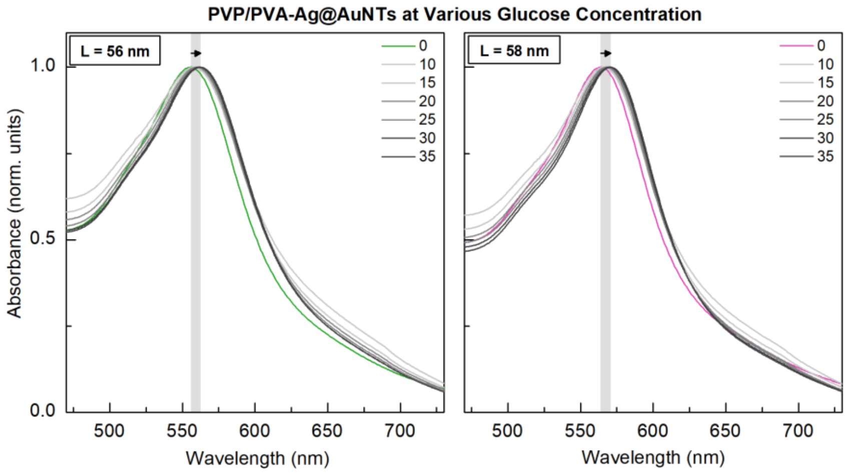

Figure S15. Normalized UV–VIS spectra of PVP/PVA-capped gold nanotriangles coated with an ultrathin layer of Ag with various edge lengths (L = 56, and 58 nm as indicated in the graph titles) at different glucose concentrations (in % w/w as indicated in legends). The plasmonic shifts are highlighted with arrows and grey areas upon the increase of glucose content.

Table S6. Determination of the bulk sensitivity (S_B) for Cit-AuNTs and PVP/PVA-Ag/AuNTs with various edge lengths. The S_B and adj. R^2 values were calculated with a linear fit function in OriginLab software.

C (Glu), % w/w	RI, RIU	mean λ_{LSPR} ± st.dev., nm					
		56 nm Cit-AuNTs	58 nm Cit-AuNTs	65 nm Cit-AuNTs	74 nm Cit-AuNTs	56 nm Ag-AuNTs	58 nm Ag-AuNTs
0	1.3322	635.0	638.0	649.0	658.0	556.0	564.0
10	1.3435	637.0 ± 0.4	640.8 ± 0.5	651.3 ± 0.2	661.5 ± 0.4	557.4 ± 0.4	565.7 ± 0.5
15	1.3487	638.4 ± 0.1	642.1 ± 0.1	653.0 ± 0.3	663.0 ± 0.2	558.6 ±0.2	566.9 ± 0.4
20	1.3542	639.3 ± 0.3	643.3 ± 0.1	653.9 ± 0.1	664.6 ± 0.1	559.8 ± 0.3	567.8 ± 0.3
25	1.3596	640.2 ± 0.2	644.3 ± 0.2	655.2 ± 0.2	665.6 ± 0.2	560.9 ± 0.2	568.8 ± 0.2
30	1.3651	641.0 ± 0.1	645.4 ± 0.2	656.2 ± 0.2	666.9 ± 0.1	561.5 ± 0.2	569.7 ± 0.1
35	1.3706	642.2 ± 0.2	646.2 ± 0.2	657.6 ± 0.2	668.1 ± 0.2	562.7 ± 0.2	570.8 ± 0.3
**S_B, nm/RIU:**		**186.6**	**224.1**	**221.4**	**274.4**	**169.5**	**173.4**
adj. R^2		0.99892	0.99730	0.99917	0.99691	0.99640	0.99911

References

[40] S. Zhou, D. Huo, S. Goines, T.-H. Yang, Z. Lyu, M. Zhao, K. D. Gilroy, Y. Wu, Z. D. Hood, M. Xie, Y. Xia, *Journal of the American Chemical Society* **2018**, 140, 11898-11901.

[54] E. Podlesnaia, A. Csáki, W. Fritzsche, *Nanomaterials* **2021**, 11.
